# Longitudinal Associations Between Fatigue and Perceived Work Ability in Cancer Survivors

**DOI:** 10.1007/s10926-018-9814-6

**Published:** 2018-11-07

**Authors:** M. D. J. Wolvers, M. C. J. Leensen, I. F. Groeneveld, M. H. W. Frings-Dresen, A. G. E. M. De Boer

**Affiliations:** 1 Coronel Institute of Occupational Health, Amsterdam Public Health Research Institute, Amsterdam UMC, University of Amsterdam, Meibergdreef 9, Amsterdam, The Netherlands; 2 Present Address: Rijnlands Rehabilitation Center, Leiden, the Netherlands

**Keywords:** Fatigue, Concentration, Perceived work ability, Cancer survivorship, Chemotherapy

## Abstract

*Purpose* To examine the associations between changes of fatigue and changes of perceived work ability in cancer survivors. Furthermore, to examine the effects of physical job demands on these associations. *Methods* Data from a feasibility study on a multidisciplinary intervention to enhance return to work in patients with cancer receiving chemotherapy was used. Fatigue (Multidimensional Fatigue Inventory) and perceived work ability (first item of the Work Ability Index) were assessed at baseline, and after 6, 12, and 18 months. Change scores (S1, S2, S3) from each assessment to the next were calculated, thus encompassing three separate time periods of 6 months. Regression analyses were used to quantify associations between change of perceived work ability and (model 1) change of general fatigue, and (model 2) change of mental and physical fatigue for each 6-month period separately. For model 2, interaction effects of perceived physical job demands were studied. *Results* A total of 89 participants were included for analysis, among which 84% with a diagnosis of breast cancer. On average, in model 1, a reduction of five points on general fatigue was associated with an improvement of one point in perceived work ability in all three 6-month periods. Model 2 showed, similarly, that change of physical fatigue (S1 and S2: B = − 0.225; *p* < .001 and B = − 0.162; *p* = .012) and change of mental fatigue (S3: B = − 0.177; *p* = .027) were significantly inversely associated with change of perceived work ability. Interaction effects were not significant. *Conclusion* The inverse, longitudinal association between fatigue and perceived work ability supports previous findings from cross-sectional studies and shows potential occupational impact of targeting fatigue in cancer rehabilitation.

**Electronic supplementary material:**

The online version of this article (10.1007/s10926-018-9814-6) contains supplementary material, which is available to authorized users.

## Introduction

Fatigue is a frequent and persistent symptom during and after cancer treatment [1]. Cancer-related fatigue is known to hamper activities of daily living and participating in social activities [2]. Fatigue does not only affect personal life, but as most cancer survivors are part of the working population, also their work life [3]. Qualitative studies showed that fatigue affects work ability [4] and hampers smooth work resumption. In line with that, fatigue has been previously pointed at as an important predictor of return to work [5, 6], of perceived work ability in long-term cancer survivors [7] and is still a common problem when cancer survivors have resumed work [8].

Cancer rehabilitation, among other things, aims at reducing fatigue to stimulate societal participation. One important aspect of societal participation that is expected to benefit from reducing fatigue is work resumption [9]. Lower fatigue is expected to result in higher self-perceived work ability, and, with that, earlier return to work. Although cross-sectional studies showed that fatigue [10] and attentional fatigue specifically (explained variance 39%) [11] are associated with low work ability, longitudinal studies on associations between fatigue and work ability are scarce. It is unknown how longitudinal changes in fatigue are associated with changes in work ability. Although we can assume that this longitudinal relationship between changes in fatigue and changes in work ability is the mechanism behind improved return to work rates after physical rehabilitation interventions, this has not been studied before.

Cancer-related fatigue is considered a multidimensional construct. The National Comprehensive Cancer Network (NCCN) definition explicitly names ‘physical, emotional, and/or cognitive tiredness’, and most multidimensional fatigue measures in the field of cancer survivorship make similar distinctions [12]. It is assumed that the extent to which patients suffer from the various aspects of fatigue, i.e. the quality of the fatigue experience, differs among individuals [13]. Although many frequently used measures in cancer populations acknowledge the multidimensionality of fatigue [14, 15], distinctions between these dimensions are often neglected when studying the development of fatigue or its consequences. Instead, general measures are used that focus on overall fatigue severity or limitations due to fatigue. Moreover, between-person differences in the experience of fatigue have rarely been explicitly studied at all.

Studying the different dimensions of fatigue can help to gain understanding of potential treatment options for fatigue, and beneficial consequences from reducing fatigue. A review on pathophysiological mechanisms of cancer-related fatigue [16] provided different explanations for mental and physical fatigue, which would require different interventions. For example, a mediation study showed that physical fatigue and not mental fatigue reduced during exercise training [17], and that reduced physical and not mental fatigue was related with improved quality of life. Similarly, it should be studied what dimensions of fatigue are related to work ability, to verify if physical training could improve work ability through lowering physical and/or mental fatigue.

As the experience of work ability is strongly related to the person’s current or most recent job, the distinction between physical and mental components could also be relevant for different job types. A review showed that poor work ability was associated with having a physically demanding job and poor physical condition [18]. Other reviews reported that physically demanding jobs and physical work load were negatively associated with work ability [3] and return to work [19]. One can argue that the work ability of employees with a physically demanding job is more hampered by physical fatigue than of employees with a job that is not physically demanding. If so, change of physical fatigue affects the work ability of such employees differently, such that (work ability of) employees in a physically demanding job benefit more from reducing physical fatigue. For this reason, we will study to what extent the association between fatigue subtypes and perceived work ability depends on the physical demands of the person’s job.

In a recent study [20], we reported that perceived work ability as well as general, physical, and mental fatigue improved in a sample of adult cancer survivors (persons who had a cancer diagnosis after their eighteenth birthday) who participated in an intervention that supported work resumption by means of vocational guidance and physical exercise training aimed at improving cardiorespiratory fitness and muscle strength. In the current paper, we will use this data in a secondary analysis to focus on the longitudinal associations between fatigue and perceived work ability.

## Research Aims

To gain insight into the potential impact of targeting fatigue in cancer rehabilitation, the current paper will study how individual changes in general, physical, and mental fatigue over time are related to changes in perceived work ability. We hypothesize that the association is significant and inverse, such that reducing fatigue is associated with improvement of perceived work ability. Secondary aims are (1) to explore cross-sectional associations of physical and mental components of fatigue, (2) to explore how perceived work ability, and physical and mental fatigue change over time, (3) to study if having a physically demanding job affects associations between fatigue change and perceived work ability change (behaves as a moderator). We hypothesize that the association between changes of physical fatigue and changes of perceived work ability is stronger in participants with physically demanding jobs compared to physically undemanding jobs.

## Methods

### Participants

Data for this study were collected as part of the AWORK project. This particular study was a pre-post study to evaluate a multidisciplinary intervention that aimed to enhance return to work. The intervention consisted of one to three consultations with an oncology occupational physician and approximately 12 weeks of exercise training, twice a week. The study involved a one-group study design without a control group. The intervention has been described in more detail elsewhere [9].

Participants were recruited in two hospitals in the Netherlands. Participants were approached by their oncologist or oncology nurse. Patients were eligible when they were aged between 18 and 60 years, had a primary diagnosis of cancer, and were being or would soon be treated with chemotherapy with curative intent. Patients were eligible when they also had been in paid employment at the time of diagnosis and were absent from work or intended to report sick before the start of treatment. Exclusion criteria were having testicular cancer, or severe mental disability, or being physically unable to perform exercise training. Ethical approval was granted from the AMC medical ethics committees. Informed consent was obtained from all individual participants included in the study. The protocol has been described previously [20].

### Procedures

Questionnaire data from assessments on four time points are used for this study. A baseline assessment (T1), and post-assessments after approximately six (T2), 12 (T3), and 18 (T4). This allows us to study the change scores of three time periods of 6 months: S1 (0–6 months), S2 (6–12 months), and S3 (12–18 months).

### Measures

Sociodemographic characteristics, among which physical demands of the participant’s job, were assessed at baseline (T1).

Fatigue was assessed with the Multidimensional Fatigue Inventory (MFI) [21] at all four time points. The MFI consists of 20 items that contribute to five subscales, measuring general fatigue (GF), mental fatigue (MF), physical fatigue (PF), reduced activity, and reduced motivation. Each item is a statement that can be rated on a scale from 1 (‘yes, that is true’) to 5 (‘no, that is not true’), leading to a score from 4 to 20, with higher scores reflecting more severe fatigue. Only the three subscales on fatigue will be used in this study. As the general fatigue scale correlates strongly with the physical fatigue scale (r = .86, [21]), both scales will be analyzed in separate models. The subscales have good internal consistency: Cronbach’s alphas were > 0.82 in patients undergoing radiotherapy [21] and > 0.95 in the current baseline assessment (GF: .951; PF: .998; MF: .999; N = 106).

Perceived work ability (after this: work ability) was assessed at all four time points with the first item of the work ability index: ‘Assume that your work ability at its best has a value of 10 points. How many points would you give to your current work ability? (0 means that you cannot currently work at all)’ [22]. This single item is considered an acceptable substitute of the full work ability index in women on long-term sick leave [23].

Physical demands of the participant’s job was assessed with one question of the Dutch Questionnaire on the Experience and Assessment of Work [24]: ‘Do you find your work very physically demanding?’ Response categories were: ‘never’, ‘sometimes’, ‘often’, and ‘always’. These scores were dichotomized, so that responding ‘sometimes’, ‘often’, or ‘always’ was operationalized as ‘perceived job as physically demanding’.

### Analytic Strategy

The analytic strategy addressed missing data handling, preliminary analysis, and a distinction between cross-sectional analyses and longitudinal analyses. All analyses were performed in SPSS, version 24. Box plots and scatter plots were generated in RStudio (RStudio Team (2016). RStudio: Integrated Development for R. RStudio, Inc., Boston, MA URL; R version 3.3.3).

### Missing Data Handling

Missing data patterns were analyzed and multiple imputations were generated to account for missing data. Data was predominantly missing as entire assessments during follow-up. 40 imputations were generated to account for missing data. The strategy for performing multiple imputations is described in more detail in Online Resource 1.

### Participants Characteristics

The sociodemographic characteristics, as well as the baseline assessments (T1) of fatigue and work ability were summarized with means (standard deviations) and percentages.

### Cross-Sectional Exploration of Physical and Mental Components of Fatigue

Levels of mental and physical fatigue on all time points (T1–T4) were visualized in four scatter plots.

### Longitudinal Trajectories of Fatigue and Work Ability

Mean trajectories and a random subset of individual trajectories were plotted for physical and mental fatigue and work ability. Individual trajectories showed that models that captured all 18 months would not acknowledge the unsteady individual trajectories. Therefore, we operationalized ‘change’ of the variables as change scores between consecutive assessments.

### Regression Models of Fatigue Change and Work Ability Change

**Fig. 1:**
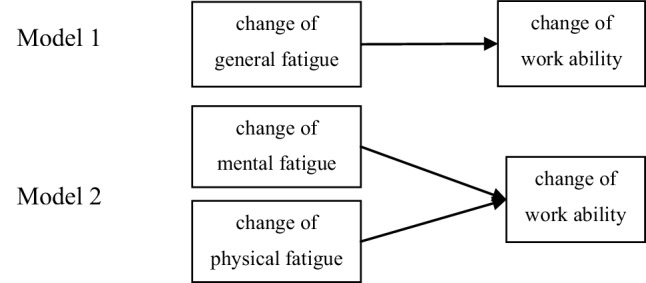
Two regression models

To address our primary research question, two linear multivariable regression models were tested with change of work ability (dWA) as dependent variable and either (1) change of general fatigue (dGF), or (2) change of both mental and physical fatigue (dMF and dPF, respectively) as independent variables (see Fig. 1).

To study the influence of perceiving a job as physically demanding to the association between fatigue change and work ability change; model 2 was supplemented with direct effects of physical demands as well as interaction effects of physical demands with mental and physical fatigue. Regression coefficients with 95% confidence intervals of physical and mental fatigue, as well as the *p*-value of the interaction effects were reported.

## Results

Of the 95 participants that were originally included and completed the intervention [20], six were excluded for the current analyses: two participants never returned the baseline questionnaire, and four did not comply with the inclusion criteria: two participants had not reported sick at work, and two participants were jobless when they completed the baseline questionnaire. A total of 89 participants were included for analyses. Assessment T1 to T4 of fatigue and work ability were complete for 83 (93%), 75 (84%), 67 (75%), and 65 (73%) of the 89 participants. Most participants were female (91%), and were diagnosed with breast cancer (84%). Participants were on average 48 ± 7 years old (mean ± standard deviation). Characteristics of the participants are reported in Table 1.

**Table 1:** Sociodemographic and clinical characteristics of 89 participants

Characteristic	n (%) or M (SD)
Sociodemographic factors	
Female	81 (91%)
Age (years)	47.9 (7.2)
Education	
Low	13 (15%)
Intermediate	30 (34%)
High	46 (52%)
Breadwinner status (n = 88)	
Sole	40 (45%)
Shared	18 (20%)
Partner	30 (34%)
Disease-related variables	
Cancer type	
Mamma	75 (84%)
Colorectal	7 (8%)
Non-Hodgkin lymphoma	5 (6%)
Other	2 (2%)
Days since diagnosis^a^ (n = 81)	83 (46)
Days since first chemotherapy^a^ (n = 84)	13 (26)
Started chemotherapy after baseline assessment (n = 84)	26 (31%)
Adjuvant chemotherapy	79 (89%)
Treatments additional to chemotherapy	
Surgery	79 (89%)
Radiotherapy	27 (30%)
Hormone treatment	11 (12%)
Radiotherapy and hormone treatment	30 (34%)
Work-related variables	
Days since first day of sick leave^a^ (n = 81)	82 (58)
Currently (partially) working	
at T1	6 (7%)
at T2 (n = 78)	46 (59%)
at T3 (n = 69)	60 (87%)
at T4 (n = 65)	55 (85%)
Type of contract (n = 88)	
Permanent employment	73 (82%)
Temporary employment	5 (6%)
Self-employed	9 (10%)
Perceived work ability (n = 86)	5.1 (2.0)
Perceived physical demands (n = 88)	
Never	41 (47%)
Sometimes	41 (47%)
Often/always	6 (8%)
Weekly working hours (n = 83)	28.7 (10.1)
Years in current employment (n = 88)	10.9 (8.4)
Years in paid employment (n = 86)	23.1 (9.5)
Works at large company (> 100 employees)	55 (62%)
Shift work/irregular service	15 (17%)
Fatigue	
General fatigue (n = 84)	12.8 (4.9)
Physical fatigue (n = 84)	12.0 (4.8)
Mental fatigue (n = 84)	11.2 (4.1)
Reduced activity (n = 84)	12.4 (4.7)
Reduced motivation (n = 84)	9.7 (3.6)

n = 89 unless stated otherwise

*M* mean, *SD* standard deviation

^a^Number of calendar days before the baseline assessment

### Cross-Sectional Exploration of Physical and Mental Components of Fatigue

**Fig. 2:**
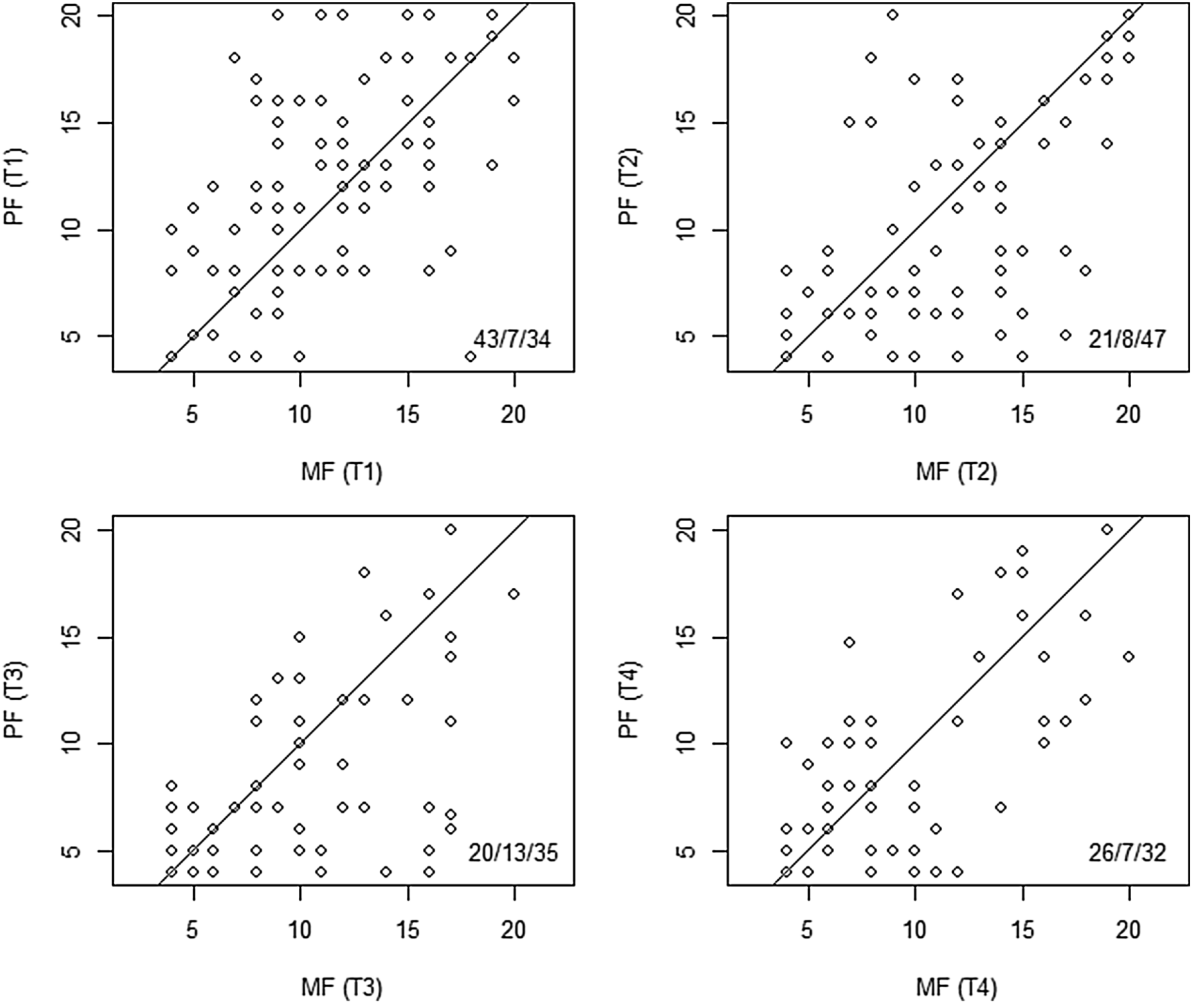
Scatter plots of physical and mental fatigue subscale scores at all four assessments. Only complete cases are shown. The MF = PF line is drawn; in the lower right corner, the number of participants above, on, and under the MF = PF line are reported

To gain insight in the heterogeneous character in the experience of mental and physical fatigue, cross-sectional scatter plots of mental and physical fatigue at all four assessments are provided in Fig. 2. In the first assessment, more participants have stronger physical than mental fatigue (43 vs. 34), whereas at T2 and T3 more participants have stronger mental than physical fatigue (21 vs. 47 and 20 vs. 35 respectively).

### Longitudinal Trajectories of Fatigue and Work Ability

**Fig. 3:**
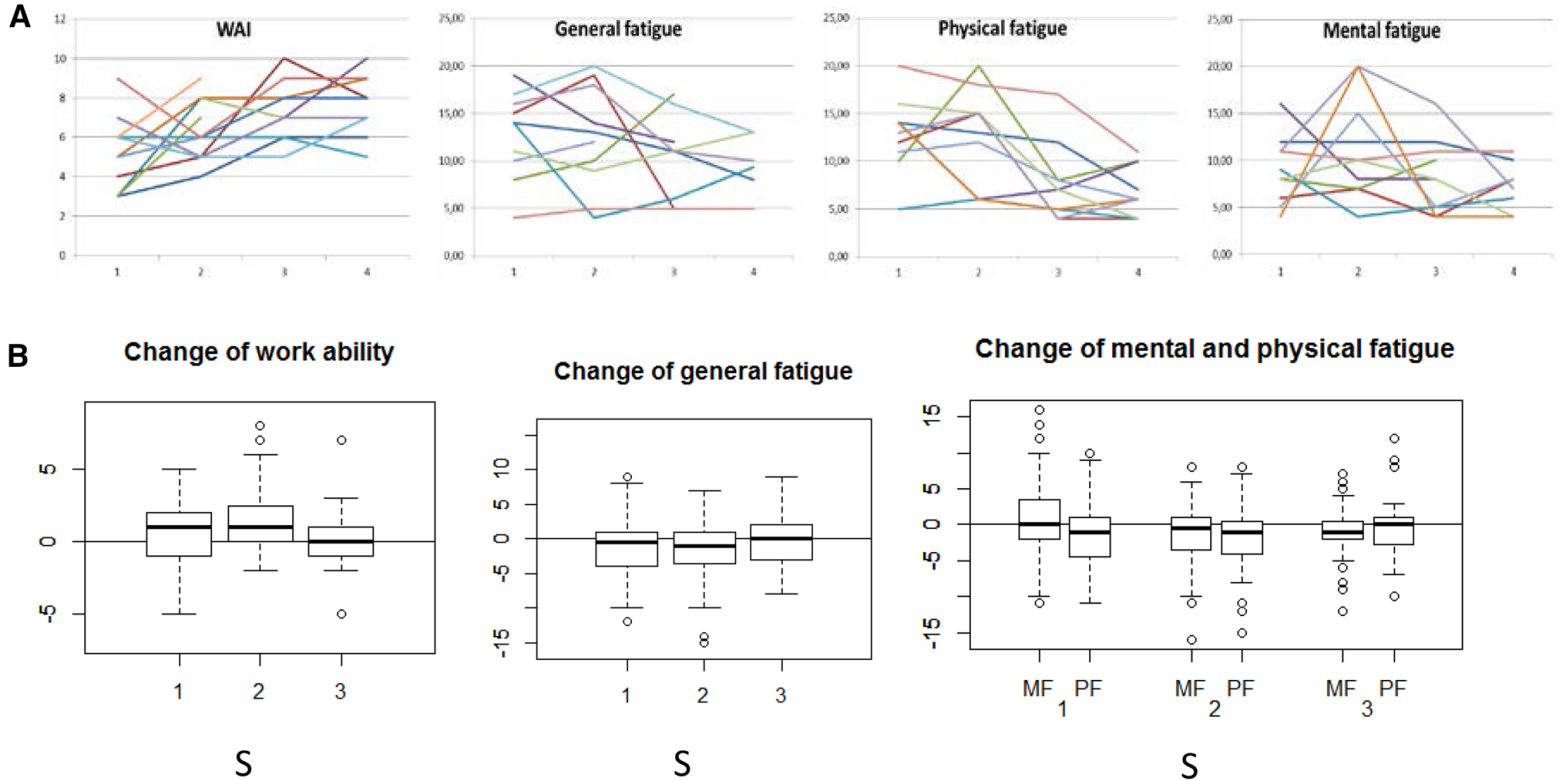
**a** Individual trajectories of work ability and fatigue from T1 to T4; **b** box plots of change scores of work ability and fatigue in 6-month periods (S) 1 to 3. *WAI* work ability

To explore how fatigue and work ability change over time, box plots of the change scores for each 6-month period as well as spaghetti plots of a subset of individual trajectories of work ability and mental, physical, and general fatigue are presented in Fig. 3. Individual trajectories of work ability and fatigue can be described as ‘tangled’; constant trajectories, as well as trajectories with large drops and jumps between the assessments are shown.

Box plots show that the median change of work ability was positive during all three 6-month periods, thus, generally, work ability improved. However, in all three periods, a portion of participants also declined. Median change of work ability as well as the variance of work ability change was smallest in the third period. Online resource 2 additionally shows mean trajectories.

Box plots show that the median change of fatigue was negative in all three 6-month periods (in each period, more participants reported a reduced fatigue than worsened fatigue), but individual change scores varied widely, especially in the first two periods.

### Regression Models of Fatigue Change and Work Ability Change

The regression analyses included 89 cases and resulted in pooled estimates of analyses on 40 imputed datasets. First, the associations between fatigue change and work ability change were studied. The analyses of model 1 show that change of work ability is inversely related to change of general fatigue in all three 6-month periods. On average, in model 1, a reduction of five points on general fatigue was associated with an improvement of one point in work ability. Inverse associations were also found in model 2, which includes mental and physical fatigue. In S1 and S2 (the first year), work ability change is associated with change of physical fatigue (B = − 0.225 and B = − 0.162, respectively), whereas in S3, work ability change is associated with change of mental fatigue (B = − 0.177). Other unstandardized regression coefficients are presented in Table 2. Scatter plots of the change scores are presented in Online resource 3. Complete case analyses are included in Online resource 1.

**Table 2:** Effects of Fatigue change on work ability change

	S	Parameter	Main effects Total sample (N = 89) B; *p*-value	Effects stratified by perceived physical job demands		
				Job perceived as never physically demanding (N = 47) B (95% CI); *p*-value	Job perceived as physically demanding (N = 42) B (95% CI); *p*-value	Interaction effect *p*-value
Model 1	1	dGF	B = − 0.269; *p* < .001			
	2	dGF	B = − 0.190; *p* < .001			
	3	dGF	B = − 0.174; *p* = .046			
Model 2	1	dPF	B = − 0.225; *p* < .001	B = − 0.208 (− 0.393 to − 0.024); *p* = .027	B = − 0.220 (− 0.382 to − 0.058); *p* = .008	*p* = .922
		dMF	B = − 0.054; *p* = .280	B = − 0.055 (− 0.186 to 0.075); *p* = .408	B = − 0.055 (− 0.198 to 0.089); *p* = .456	*p* = .995
	2	dPF	B = − 0.162; *p* = .012	B = − 0.190 (− 0.375 to − 0.004); *p* = .045	B = − 0.129 (− 0.294 to 0.035); *p* = .123	*p* = .622
		dMF	B = − 0.096; *p* = .169	B = − 0.152 (− 0.328 to 0.023); *p* = .089	B = − 0.023 (− 0.209 to 0.163); *p* = .806	*p* = .283
	3	dPF	B = − 0.086; *p* = .254	B = − 0.164 (− 0.385 to 0.057); *p* = .145	B = − 0.039 (− 0.237 to 0.159); *p* = .697	*p* = .385
		dMF	B = − 0.177; *p* = .027	B = − 0.199 (− 0.371 to − 0.028); *p* = .023	B = − 0.162 (− 0.423 to 0.098); *p* = .219	*p* = .802

Pooled data of 40 imputations is presented. Unstandardized regression coefficients are presented for all three 6-month periods separately. Perceived physical demands of the job was assessed at baseline. A regression coefficient B = − 0.200 means that on average, a decrease of five points in fatigue (scale: 0–20) is associated with an increased work ability of one point (scale: 0–10)

*CI* confidence interval, *S* 6-month period, *dGF* change of general fatigue, *dPF* change of physical fatigue, *dMF* change of mental fatigue

To study if having a physically demanding job affects associations between fatigue changes and perceived work ability changes (behaves as a moderator), differences in these associations due to perceived physical job demands were studied. The data did not provide support for a significant interaction effect (the smallest *p*-values was 0.283), which indicates that differences in perception of physical job demands do not correspond with differences in the association between fatigue and work ability. Unstandardized regression coefficients are presented in Table 2.

## Discussion

In this paper, longitudinal associations between different components of fatigue and work ability were studied to create a better understanding of potential beneficial effects of cancer rehabilitation. We hypothesized that both are inversely related, such that reduced fatigue is associated with improved work ability, which was supported by the statistical analyses.

### Cross-Sectional Exploration of Mental and Physical Components of Fatigue

The cross-sectional plots showed large differences between individuals; the levels of physical and mental fatigue differed and were widely scattered as all corners and quadrants were occupied. It should be noted that this sample is specific in the way that it considers only patients who received chemotherapy as well as an exercise intervention for fatigue. Results cannot directly be extrapolated to courses of mental and physical components of fatigue after chemotherapy or care as usual.

Our results match that of a previous study [13], which concluded that mental and physical fatigue were strongly associated in the general population, but barely in cancer survivors and not at all in advanced cancer patients. We share the conclusion of De Raaf et al. that, in cancer survivors, there might be subgroups: physically fatigued, only mentally fatigued, and both physically and mentally fatigued. As underlying mechanisms differ [16], it makes sense to distinguish (in categories or more gradually) between these subgroups when describing fatigue, but also when considering appropriate treatment.

Recently, a study was published that performed latent class analysis of dichotomizations of all five subscales of the MFI in 1183 long-term colorectal cancer survivors [25]. In these analyses, individual dimensions of fatigue did not distinguish between classes (which could be expected if these dimensions are actually independent), but rather the average level of (general, physical and mental) fatigue and the extent to which activity and motivation were reduced (‘distress’). However, this sample consisted of older, and more often male participants, with different diagnoses and treatments compared to the current sample, and the analysis included general fatigue, reduced activity, and reduced motivation subscales in addition to the physical and mental fatigue subscales. As such, these results do not oppose a distinction of aforementioned subgroups of fatigue.

### Longitudinal Trajectories of Fatigue and Work Ability

The longitudinal plots showed that fatigue trajectories were unsteady and showed large inter-individual differences. This can be explained by the variety of events that took place during the study period of which they are known or expected to influence fatigue: the initiation (31%) and continuation (69%) of chemotherapy, radiotherapy (30% of the participants), finishing chemotherapy (all participants), and participation in an intervention aimed at reducing fatigue (all participants). These results show that a description of mean trajectories really does not reflect the fatigue trajectory of any individual. They show furthermore that linear models are not sufficient to capture this variety of trajectories of fatigue in cancer patients who receive or have recently finished chemotherapy, and participate(d) in an exercise intervention.

Comparison of these results, which are reflective of the exercise intervention all participants were exposed to, unfortunately, is limited to observational studies. Also on a smaller time-scale and in the absence of an exercise intervention, large variability in trajectories has been reported for patients during and after cancer treatment. Spaghetti plots showed large inter-individual variability of trajectories of morning and evening fatigue of breast cancer patients who underwent radiotherapy [26]. Also, clinically distinct fatigue trajectories were found in persons diagnosed with and treated for colorectal cancer [27].

Another observational study found that trajectories of fatigue in the 18 months after a diagnosis of colorectal cancer were best captured in four distinct categories. Three categories consisted of quite stable trajectories, and one showed a steep reduction of fatigue during the initial 7 months after diagnosis [28]. It should be noted that it was not an intervention study and incorporated one fewer assessment. However, in this particular study, a response shift was observed [28], which means that a participant’s internal concept of fatigue severity revised, such that a similar experience of fatigue would be assigned a lower score when it was assigned post-treatment (judged along post-treatment internal standards of fatigue) compared to pretreatment. This mechanism leads to an underestimation of the deterioration of fatigue during treatment and post-treatment fatigue levels. Although the fatigue measure for which this response shift was observed differs from the MFI that was used in the current study, a similar mechanism could exist in the current sample, which is of particular ‘risk’ for a response shift due to the high peak of fatigue that is associated with chemotherapy. Such a response shift would mostly affect fatigue estimates of S1 and would make regression estimates (even) less comparable between the time periods.

### Regression Models of Fatigue Change and Work Ability Change

Model 1 showed that change of general fatigue is associated with change of work ability during all three 6-month periods such that five points reduction of general fatigue is associated with one point improvement of work ability. Similar effect sizes were found in model 2: change of work ability was associated with change of physical fatigue in the first year, and with change of mental fatigue in S3. Although these results uphold findings in cross-sectional studies and could be explained by a causal relationship, a number of alternative explanations would provide similar results. For example, some unobserved factor works as a strong confounder for these effects, such as general health or severity of comorbid conditions. Comparison to existing literature was not possible, as no comparable studies are yet available. One study might be relevant in this perspective though. Very weak support for a relation between reduction of fatigue and improvements of work ability was found previously in patients who suffered from chronic cancer-related fatigue and participated in an intervention aimed at reducing fatigue [29]. Analysis of the follow-up period provided weak support that such an effect was not present. Our last hypothesis, which stated that having a physically demanding job results in a stronger inverse association between change of physical fatigue and change of work ability, was not supported by the data: effect differences due to perceived physical job demands were statistically nonsignificant and the regression coefficients of S3 could even suggest an effect opposite to our hypothesis. In any case, there is no reason to refute our null-hypothesis of no difference between physically demanding and undemanding jobs. Notably, the dichotomization of perceived physical demands was necessary due to the limited sample size, but the resulting contrast at hand could have been sub-optimal. Comparison of the categories ‘sometimes’ and ‘less often’ perceived as physically demanding versus ‘often’ or ‘always’ perceived as physically demanding might be a better parallel with other dichotomous variables on physical job demands [30, 31]. As the formulation of the question is very general, it is unclear what (types of) physical demands or strains are referred to by participants when considering ‘perceived physical job demands’, and whether these are demands that are relevant for work ability and employment.

### Strengths and Limitations

Strengths of this study are the long follow-up period of 18 months, allowing the analysis of three separate time periods of 6 months, and the focus on individual change scores instead of cross-sectional associations. Also, the distinction between mental and physical fatigue is novel in this context.

Our study has several limitations. As stated, a response shift of fatigue could have biased the estimates of associations during S1. Also, not including potentially confounding factors is addressed here as a limitation. Overall health status, functional capacity and sickness absence have not been eliminated as underlying mechanisms for the effects that were observed. For example, one could argue that the perception of physical job demands is influenced by the well-being or physical condition of the participant. As such, it would be strongly confounded. However, bias is expected to be limited because, in other populations, the perception of job demands was independent from well-being cross-sectionally [32], and independent of health status, physical activity [33], and fatigue [31] 1 year previously. Additionally, especially in S1, consultations with the OOP could be responsible for improvements in work ability. This study did not have a design that could estimate such direct effects of the intervention.

### Future Research and Recommendations

Although this work provides some insight in trajectories of fatigue and its association with change of work ability, the time between assessments should be reduced drastically to understand and to capture intra-individual variation in both constructs.

Although no causality can be derived from this study, this result is worth considering as an argument for the benefits of fatigue intervention as part of (occupational) cancer rehabilitation. Considering the strong predictive value of perceived work ability for return to work, perceived work ability would be a very useful and relevant outcome for effectiveness studies in cancer rehabilitation that somehow focuses on reducing or limiting fatigue.

## Conclusion

A reduction of fatigue is generally associated with an improvement of work ability, such that five points on general fatigue are associated with one point improvement of work ability. We found no effect of physical demands on this association. The inverse, longitudinal association between fatigue and work ability supports previous findings from cross-sectional studies and shows potential occupational impact of targeting fatigue in cancer rehabilitation.

## Electronic supplementary material

Below is the link to the electronic supplementary material.

Supplementary material 1 (DOC 120 KB)
