# Supplementary material for: Longitudinal Associations Between Fatigue and Perceived Work Ability in Cancer Survivors
Source: J Occup Rehabil. 2018 Nov 7;29(3):540–9. doi: 10.1007/s10926-018-9814-6 (PMC6675773; doi:10.1007/s10926-018-9814-6)
Supplement: Supplementary file 1 — Supplementary material 1 (DOC 120 KB) [file 10926_2018_9814_MOESM1_ESM.doc]

# Online resource 1

*Article title:* Longitudinal associations between fatigue and work ability in cancer patients

*Authors:* Wolvers MDJ, Leensen MCJ, Groeneveld IF, Frings-Dresen MHW, De Boer AGEM

*Journal:* Journal of Occupational Rehabilitation

*Corresponding author*: A.G.E.M. de Boer, Academic Medical Center, department: Coronel Institute of Occupational Health, Amsterdam Public Health research institute, Amsterdam, the Netherlands (a.g.deboer@amc.uva.nl | +31 20 566 5323)

*Content:* This online resource provides supplementary information on generating multiple imputations and on a complete cases analyses for Models 1 and 2 of which the pooled imputed results are provided in the manuscript.

## Multiple imputations

As only 54% of the cases had no missing values on any of the fatigue and work ability measures, multiple imputations were generated to prevent that cases are discarded due to missing data. Fully conditional specification[[1]](#footnote-2) (also known as chained equations or MICE) in SPSS, version 24 was used to generate these imputations. The predictive mean matching (PMM) method was used for all (scale) variables, following recommendations from Marshall et al. (2010). [[2]](#footnote-3) PMM is similar to regression methods, but relies less on the parametric assumptions of the imputation models as the observed value that is closest to the value drawn by the imputation model is imputed.

First, 20 imputed datasets were generated. After convergence of the models was visually inspected using the trace plots of two imputations up to 500 iterations each, the number of iterations was set at 50. A total of 40 imputed datasets were generated, following recommendations to prevent loss of power[[3]](#footnote-4) and to have optimal reproducibility. [[4]](#footnote-5)

### Imputed variables

Missing data of work ability and fatigue at T1, T2, T3, and T4, and physical job demands at T1 were imputed. Fatigue data were imputed at scale level, so as subscale sum scores. Although item-level imputation is a preferred method compared to scale-level imputation because of superior efficiency[[5]](#footnote-6), this approach was not feasible due to the small sample size[[6]](#footnote-7).

### Auxiliary variables

As the inclusion of auxiliary variables in the imputation regression models can reduce bias[[7]](#footnote-8), we included the following variables as predictor variables in the imputation models for all imputed variables: perceived mental and physical work ability (as scalar, range from 1 to 5), job self-efficacy (Lagerveld et al.), and the sum scores of the fatigue and health subscale of the EORTC-QLQ-30[[8]](#footnote-9) (all at T1 to T4). Furthermore, age, gender, education level, peak oxygen consumption at T1 and T2 (assessed on a bicycle ergometer by a sports physician), and time since first chemotherapy.

### Pooling

Analyses were performed on each dataset and pooled results are reported in the main text.

To get an understanding of the influence of ignoring cases with missing data, Table 1 presents the correlations matrix at the final assessment for complete cases only and for pooled data. Similarly, complete case analysis for the main research question is provided in Table 1.

Table 1. Cross-sectional correlates at the final follow-up assessment (T4) on complete cases and imputed data

|  | Work ability | General fatigue | Physical fatigue | Mental fatigue |
| --- | --- | --- | --- | --- |
| Work ability | 1 | .706 | .690 | .718 |
| General fatigue | .494 | 1 | .848 | .652 |
| Physical fatigue | .511 | .752 | 1 | .620 |
| Mental fatigue | .582 | .483 | .439 | 1 |

Note: the lower (left) triangle presents the pooled estimates of multiple imputations (N = 89), the upper (right) triangle presents the estimates of the complete cases only (N = 65). All correlations have a *p*-value below .001.

Table 2. Complete case analysis of multiple imputations for the regression coefficients

|  |  |  | Complete cases | | Imputed data ^a^ | |
| --- | --- | --- | --- | --- | --- | --- |
|  | S |  | n | Regression coefficients | Regression coefficients | Fraction missing information |
| Model 1 |  |  |  |  |  |  |
|  | 1 | dGF | 70 | B = -0.309; *p* < .001 | B = -0.269; p < .001 | .302 |
|  | 2 | dGF | 62 | B = -0.248; *p* < .001 | B = -0.190; *p* < .001 | .246 |
|  | 3 | dGF | 55 | B = -0.217; *p* = .005 | B = -0.174; *p* = .046 | .549 |
| Model 2 |  |  |  |  |  |  |
|  | 1 | dPF | 70 | B = -0.227; *p* <.001 | B = -0.225; *p* < .001 | .287 |
|  |  | dMF |  | B = -0.036; *p =* .464 | B = -0.054; *p* = .280 | .233 |
|  | 2 | dPF | 62 | B = -0.207; *p* = .001 | B = -0.162; *p* = .012 | .347 |
|  |  | dMF |  | B = -0.095; *p* = .114 | B = -0.096; *p* = .169 | .495 |
|  | 3 | dPF | 55 | B = -0.100; *p =* .124 | B = -0.086; *p* = .254 | .528 |
|  |  | dMF |  | B = -0.190; *p =* .005 | B = -0.177; *p* = .027 | .591 |

Note: fraction of missing information is a measure of efficiency of the imputation model. ^a^ Pooled data are presented for N = 89. Abbreviations: S (semester), dGF (change of general fatigue), dPF (change of physical fatigue), dMF (change of mental fatigue).

# Online resource 2

*Article title*: Longitudinal associations between fatigue and work ability in cancer patients

*Authors*: Wolvers MDJ, Leensen MCJ, Groeneveld IF, Frings-Dresen MHW, De Boer AGEM

*Journal:* Journal of Occupational Rehabilitation

*Corresponding author*: A.G.E.M. de Boer, Academic Medical Center, department: Coronel Institute of Occupational Health, Amsterdam Public Health research institute, Amsterdam, the Netherlands (a.g.deboer@amc.uva.nl | +31 20 566 5323)

*Content:* This online resource provides plots of the mean trajectories of fatigue and work ability in addition to the box plots and individual trajectories in the main manuscript.


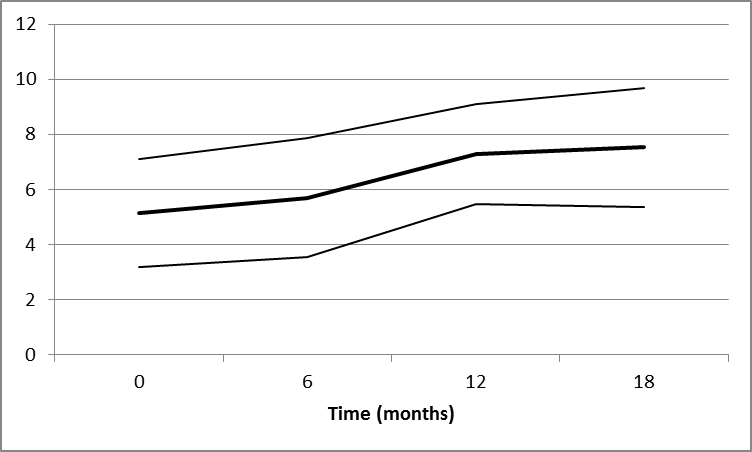


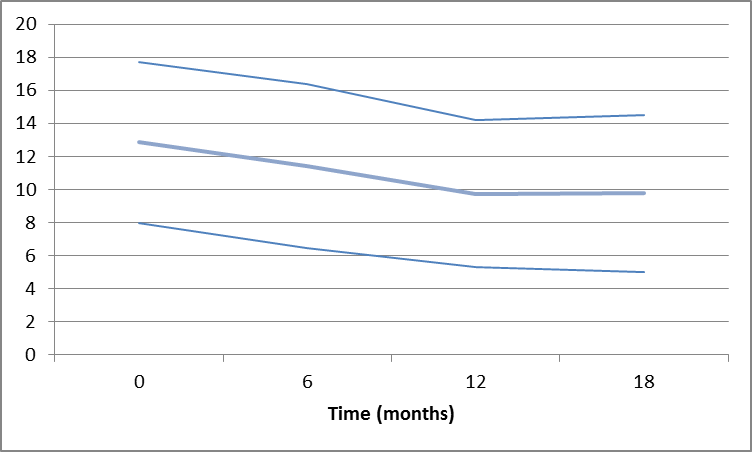


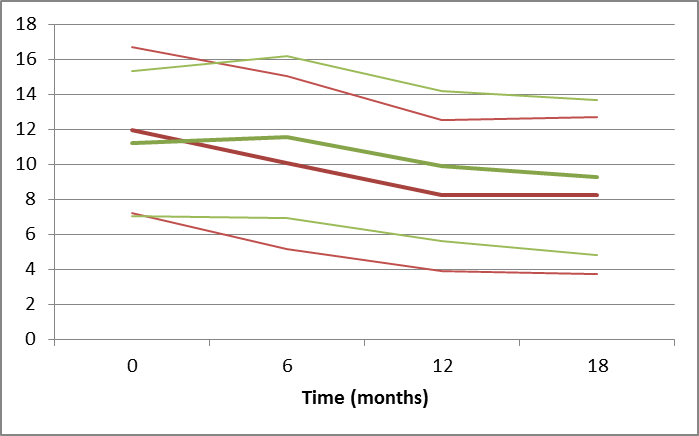


Figure 1. Mean (+/- standard deviation) trajectories and box plots of the change scores of work ability (top), general fatigue (middle), physical (red) and mental (green) fatigue

# Online resource 3

*Article title:* Longitudinal associations between fatigue and work ability in cancer patients

*Authors:* Wolvers MDJ, Leensen MCJ, Groeneveld IF, Frings-Dresen MHW, De Boer AGEM

*Journal:* Journal of Occupational Rehabilitation

*Corresponding author*: A.G.E.M. de Boer, Academic Medical Center, department: Coronel Institute of Occupational Health, Amsterdam Public Health research institute, Amsterdam, the Netherlands (a.g.deboer@amc.uva.nl | +31 20 566 5323)

*Content:* This online resource provides proportions of participants for whom both fatigue and work ability changed, as well as scatter plots of the change scores of fatigue and work ability.

## Change scores: frequencies and scatter plots

To estimate to what extent the direction of associations among fatigue and work ability were constant within individuals, we established how many of the participants with changed fatigue (|dF| > 1) experienced a work ability change in the opposite direction (negative association) or no change of work ability ( |dWA| ≤ 1 ).

The consistency of the hypothesized negative association between physical/mental fatigue and work ability within participants was studied. For general fatigue, in 83.4% of the participants with changed fatigue, the work ability remained either stable or improved in all three semesters. For mental and physical fatigue, this percentage is 83.3% and 79.6% respectively. Results for each semester separately are presented in Table 3.

Table 3. Percentages of associations between fatigue change and work ability change

|  | General fatigue | | | Physical fatigue | | | | Mental fatigue | | |
| --- | --- | --- | --- | --- | --- | --- | --- | --- | --- | --- |
|  | S1 | S2 | S3 | S1 | S2 | S3 | S1 | | S2 | S3 |
|  | N (%) | N (%) | N (%) | N (%) | N (%) | N (%) | N (%) | | N (%) | N (%) |
| No fatigue change ^a^ | 27.2 (31%) | 28.8 (32%) | 28.2 (32%) | 27.5 (31%) | 35.6 (40%) | 32.8 (37%) | 20.3 (23%) | | 32.3 (36%) | 32.7 (37%) |
| Fatigue change | 61.8 (69%) | 60.2 (68%) | 60.8 (68%) | 61.6 (69%) | 53.5 (60%) | 56.3 (63%) | 68.8 (77%) | | 56.7 (64%) | 56.4 (63%) |
| Inverse association ^c^ | 29.8 (48%) | 24.2 (40%) | 21.8 (36%) | 27.3 (44%) | 25.3 (47%) | 20.0 (36%) | 23.5 (34%) | | 26.8 (47%) | 21.2 (38%) |
| Positive association ^d^ | 3.7 (6%) | 7.3 (12%) | 7.3 (12%) | 4.0 (6%) | 5.1 (10%) | 7.3 (13  %) | 7.8 (11%) | | 5.1 (9%) | 6.9 (12%) |
| No change of work ability ^b^ | 28.3 (46%) | 28.7 (48%) | 31.7 (52%) | 30.2 (49%) | 23.1 (43%) | 29.0 (52%) | 37.5 (55%) | | 24.8 (44%) | 32.7 (58%) |

Note: frequencies (N) are not integers as they are pooled results. ^a^|dF| ≤ 1. ^b^ |dWA| ≤ 1. ^c^ dF/dWA < 0 &|dWA| > 1.^d^ dF/dWA > 0 & |dWA| > 1.

Table 3 shows that in 34 to 48% of the cases in which fatigue change (general, physical or mental fatigue) of at least two points was observed, additionally change of work ability of at least two points in the inverse direction was observed. In only 6 to 13% of the cases in which fatigue was observed to improve, work ability declined or vice versa.

Scatter plots of all change scores are presented in Figure 2.


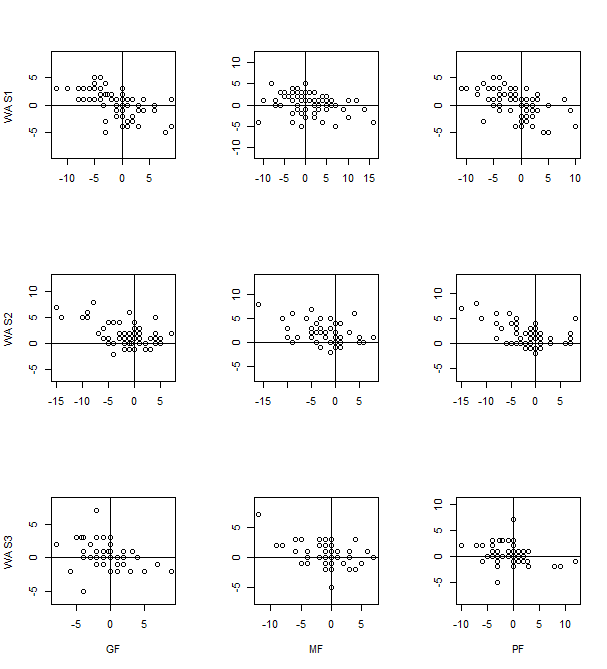


Figure 2. Scatter plots of changes scores of work ability with change scores of general, mental, and physical fatigue (GF, MF, and PF respectively) for all three semesters

1. Van Buuren 2007 Stat Methods Med Res **16**: 219–242. [↑](#footnote-ref-2)
2. Marshall, Altman, and Holder 2010. BMC Med Res Methodology **10**:112. [↑](#footnote-ref-3)
3. Graham, Olchowski, and Gilreath 2007. Prev Sc **8**: 206-13. [↑](#footnote-ref-4)
4. White, Royston, and Wood 2011. Statistics in Med **30**: 377-99. [↑](#footnote-ref-5)
5. Gotschall et al 2012. Multivar Beh Res **47**: 1-25. [↑](#footnote-ref-6)
6. The sample size should (greatly) exceed the number of imputed variables, which would be confined by having to impute 48 additional variables for item-level compared to scale-level imputation ((15 items - 3 sum scores) x 4 assessments). [↑](#footnote-ref-7)
7. Azur et al. 2012. Int J Methods Psychiatr Res **20**: 40-9. [↑](#footnote-ref-8)
8. Aaronson et al. 1993. J Nat Canc Inst **85**: 365-76. [↑](#footnote-ref-9)
